# A Heterogeneous Edge-Fog Environment Supporting Digital Twins for Remote Inspections

**DOI:** 10.3390/s20185296

**Published:** 2020-09-16

**Authors:** Luiz A. Z. da Silva, Vinicius F. Vidal, Leonardo M. Honório, Mário A. R. Dantas, Milena Faria Pinto, Miriam Capretz

**Affiliations:** 1 Department of Electrical Engineering, Federal University of Juiz de Fora, Juiz de Fora 36036-900, Brazil; luiz.zillmann@engenharia.ufjf.br (L.A.Z.d.S.); vinicius.vidal@engenharia.ufjf.br (V.F.V.); 2 Department of Computer Science, Federal University of Juiz de Fora, Juiz de Fora 36036-900, Brazil; mario.dantas@ice.ufjf.br; 3 Department of Electronics Engineering, Federal Center for Technological Education of Rio de Janeiro, Rio de Janeiro 20271-110, Brazil; milena.pinto@cefet-rj.br; 4 Department of Electrical and Computer Engineering, Faculty of Engineering, Western University, London, ON N6G 1G8, Canada; mcapretz@uwo.ca

**Keywords:** fog-edge computing, distribuited 3D reconstruction, heterogeneous environment, digital twins, remote inspection

## Abstract

The increase in the development of digital twins brings several advantages to inspection and maintenance, but also new challenges. Digital models capable of representing real equipment for full remote inspection demand the synchronization, integration, and fusion of several sensors and methodologies such as stereo vision, monocular Simultaneous Localization and Mapping (SLAM), laser and RGB-D camera readings, texture analysis, filters, thermal, and multi-spectral images. This multidimensional information makes it possible to have a full understanding of given equipment, enabling remote diagnosis. To solve this problem, the present work uses an edge-fog-cloud architecture running over a publisher-subscriber communication framework to optimize the computational costs and throughput. In this approach, each process is embedded in an edge node responsible for prepossessing a given amount of data that optimizes the trade-off of processing capabilities and throughput delays. All information is integrated with different levels of fog nodes and a cloud server to maximize performance. To demonstrate this proposal, a real-time 3D reconstruction problem using moving cameras is shown. In this scenario, a stereo and RDB-D cameras run over edge nodes, filtering, and prepossessing the initial data. Furthermore, the point cloud and image registration, odometry, and filtering run over fog clusters. A cloud server is responsible for texturing and processing the final results. This approach enables us to optimize the time lag between data acquisition and operator visualization, and it is easily scalable if new sensors and algorithms must be added. The experimental results will demonstrate precision by comparing the results with ground-truth data, scalability by adding further readings and performance.

## 1. Introduction

Equipment maintenance is part of several engineering environments, from power plants to industrial production lines. This task accomplishment demands skilled professionals, being part of the staff or outsourced. Therefore, several courses and training are required to offer the employee the appropriate skills. An interesting approach is to provide a detailed and safe environment through the virtual reality [1,2]. There are several different methods of creating a virtual environment and the three-dimensional objects which compose it. Traditional computer-aided design software method (e.g., AutoCAD and SolidWorks) often demands skilled labor from designers, and a long time for development. Nowadays, however, there are more sophisticated methods that utilize images captured from an object to reconstruct it, called three-dimensional reconstruction, or just 3D reconstruction [3]. This method can be seen in the literature being used in several applications, from medical to robotics and 3D games development [4,5,6,7]. As an example, self-driving vehicles use the images captured from multiple cameras to provide 3D mapping and 3D obstacle detection to navigate in complex environments [8]. Häne et al. [9] employed several low-cost cameras to achieve 360-degree coverage and an accurate multi-view stereo. Their software stack is based on the Robot Operating System (ROS) framework and they applied multi-view geometry and 3D geometric mapping to navigate in multi-level structures. A similar work is presented in Cui et al. [10], where the authors presented a real-time dense geometric mapping algorithm for 3D scene perception for self-driving vehicle. Milella and Reina al. [11] implemented a 3D scene reconstruction from a multi-baseline stereo frame, classifying the scenes into navigable and non-navigable areas.

Regarding 3D reconstruction methods, the most commonly found in the literature are those based on multiple views. In other words, where views are captured from a scene or an object, as an offline method, also called Multi-View Stereo (MVS). Other commonly seen methods are the real-time dense Simultaneous Localization and Mapping (SLAM) [12,13]. The first one, even producing exciting results in the point cloud quality, was not conceived for real-time applications and is very heavy and node in most cases. On the other hand, the second kind is based on SLAM, proposing a motion tracking in real-time while also mapping the environment. It generates a dense reconstruction through the frames sequentially acquired, and also demands the use of a GPU in many cases [14,15,16].

The number of devices required in these reconstruction processes could be exceptionally large and with different types of architectures. Therefore, an orchestration is necessary to gather [17] information from these devices and to produce a synchronized differentiated result from these nodes. This issue is common in IoT architectures, where many devices with limited computational power are distributed along with the network [18,19,20]. Those limited devices need strategies to allow the tasks distribution without affecting the data’s capability. As mentioned in the case study presented in [21], edge computing platforms can be classified as (i) resource-rich servers deployed at the edge; (ii) heterogeneous edge nodes and edge-cloud federation; and (iii) based upon their classes and architectures.

Note that 3D reconstruction through cloud-based images faces challenges, such as large size and number of photos to upload and delay. A prominent solution is the use of heterogeneous edge nodes deployed close to end-users to provide cloud-computing capabilities. Heterogeneous edge nodes are useful architectures to tackle issues where the configuration requires different kinds of devices to provide successful results. Usually, the sensor’s devices that compose this type of architecture can be seen as IoT devices, where many different sensors with limited resources are connected to a network. This is the case where devices, such as robots and cameras, are utilized to gather data to process and provide accurate information to an application (e.g., 3D reconstruction).

Therefore, this paper presents a research contribution workthat is characterized by heterogeneous edge nodes, as a computational environment, to provide data to a dense 3D reconstruction application. 3D online reconstructions using two sets of moving cameras are proposed to demonstrate methodology effectiveness. In this scenario, cameras are positioned at the edge to gather the data and perform the initial processing steps. Then, the data are forwarded to the fog using Robot Operating System (ROS) publisher-subscriber structure. More sophisticated data processing is performed in the fog, enabling the development of complex sensor architectures with limited resources at the edge, overcoming issues in state-of-the-art. Our experiments indicated an interesting set of aspects and issues considering this heterogenous edge configuration. This is a particularly relevant contribution, since, for instance, IoT software application developers are facing more heterogeneous and complex environments.

The rest of this paper is organized as follows. In Section 2, we present concepts and related work to 3D reconstruction and heterogeneous edge environments. The 3D reconstruction proposed application is described in Section 3. In Section 4, the experimental scenarios and results are explained. Finally, the conclusions and future work are presented in Section 5.

## 2. 3D Reconstruction, Heterogeneous Edge Environments, and Related Work

This section presents concepts related to the 3D reconstruction, in addition to heterogeneous edge environments. It also presents related works providing a view of other efforts. The goal is to provide in parallel technical aspects and challenges. This is especially relevant to illustrate other projects related to reconstruction, and the computational paradigm adopted to support our research proposal.

### 2.1. 3D Reconstruction

3D reconstruction can be conceived in several directions. One method is based on multiple images with different points of view to obtain the depth measurements, generate point clouds, and track camera position and orientation in a scene. This method is coined as Structure from Motion (SfM), which generally works by generating and tracking sparse feature-based models from different points of view [12]. Schönberger and Frahm [13] present in their work a review and an algorithm that improves the incremental SfM for unordered images. The authors claim that this is the most popular method for the problem. The algorithm, with an open-source version (called Colmap [22]), aims to improve robustness, accuracy, completeness, and scalability obtained through the application of scale invariante feature transform (SIFT) descriptors as features to be matched [23]. Their results are interesting when compared with state-of-the-art methods, achieving detailed reconstructions, suggesting that the usage of features would help to complete a better reconstruction [13,24,25]. However, the time consumption is a constraint even using facilities such as GPU and clusters [26]. This can be explained since the dense reconstruction step is slower than the sparse step and other methods, such as dense SLAM based reconstruction.

Regarding the monocular Simultaneous Localization and Mapping (SLAM) methods, they were developed as an alternative with great focus in real-time solutions. In this subgroup, there are several techniques offering different features. Their approach follows basically the same steps: acquiring consecutive images; aligning those images; detecting loop closures; and global model alignment [27]. MonoSLAM [28] was the first to achieve real-time localization and mapping, using just one camera and obtaining a sparse model as a result. However, the first live dense reconstruction system, working also with a single camera, was coined as Dense Trancking and Mapping (DTAM), by Newcombe et al. [14]. These methods adopted the overlapped images to generate and minimize a cost function based on photometric error. It aligns a dense model from a whole image to a given dense model of a scene. The goal is to track the camera motion, updating and expanding the global model densely.

The previous results inspired other works not using the single monocular camera approach anymore, but RGB-D or stereo cameras. The RGB-D sensor, as the name suggests, is composed by an RGB camera and a depth sensor, and is based on two types of technology: the Structured Light Sensor, with its main examples being the Microsoft Kinect, Orbbec Astra, and ASUS Xtion PRO, and the more modern type, the time-of-flight, such as Microsoft Kinect II [15,29,30]. On the other hand, stereo cameras are composed of two RGB cameras, using a stereo disparity technique to estimate depth instead of measuring it [31,32]. Therefore, it is possible to use stereo cameras (or a stereo camera rig) for 3D reconstruction, as it is reported in [33].

Works in the literature clearly show advancements with RGB-D cameras, as is the Kinect Fusion reconstruction system of Newcombe et al. [15], which was the first to densely reconstruct in real-time using a Kinect sensor and a GPU parallel processing algorithm. Its pipeline is divided into a first step of capturing the sensor measurements, generating a vertex and normal map from the depth map. Next, the Iterative Closest Point (ICP) algorithm is used to refine the camera pose for the current frame, being the result integrated into the global model through Truncated Signed Distance Function (TSDF). Finally, the TSDF is used on the generated surface, producing an estimated frame to be used as a comparison to the next image acquisition.

In the same direction, there is the Elastic Fusion (EF) algorithm, produced by Whelan et al. as a more map-centric approach [34]. Similar to the Kinect Fusion, this one utilizes an RGB-D camera and Compute Unified Device Architecture (CUDA) parallel programming generating a surfel-based reconstruction in real-time [35,36]. These surfel elements compose the 3D model and carry the information of pose, normal, RGB colors, weight, element radius, and timestamps for the process initialization, and the last frame acquired. The pose estimation is done through the minimization of a cost function. This is composed of an element of photometric error carrying the RGB information, and another of geometric error, carrying the depth map information—thus obtaining the motion parameters for the current frame. They also applied an active or inactive strategy to determine whether the surfel is going to be used for tracking and fusion or not. For the global loop closure, it stores a randomized fern database for further application in place recognition. If a match is detected, the views are registered together and, if they are globally and geometrically consistent, the map is deformed non-rigidly. In [12], it is stated that this method is robust and accurate, in the tracking and mapping steps when compared with the other state-of-the-art methods.

Similar principles are found in the Bundle Fusion [37], which also uses the cost function of EF, but with the addition of feature-based parameters obtained from a sparse cloud to obtain the pose alignment. As it is reported in [12], they tried to improve the localization problems found in the previous methods. This is achieved by the independence of temporal coherence, making a robust re-localization system even with sensor occlusion or sudden movements. However, it is also mentioned that, although it has superior performance, the method still suffers when facing sudden movements or different paths, which generates misalignments in the registered model. As shown before, there is a global misalignment problem due to localization estimation errors. As a result, it is also valid to cite the work from Ibragimov and Afanasyev [38]. The authors claimed that the odometry results obtained by the stereo camera ZED were reasonable when compared to a LIDAR sensor, and superior when also compared with another state-of-the-art SLAM technique, ORB-SLAM, which is feature-based, fast, and accurate [39,40]. This result suggests that the odometry obtained with stereo cameras can be used as an alternative to mitigate errors in global model alignment.

Desspite superior performance on Visual Odometry (VO), other works present the relevant errors in many applications when comparing point clouds’ accuracy generated from structured light and stereo sensors. In [41], several cameras are analyzed and compared in length and area accuracy in terms of pixel coefficients. Even though the ZED camera presents good and sometimes the best results in outdoor applications, there are more flaws and inaccuracies in close objects, with depth deviations that lead to wrong measurements of volume and area. Note that some works use both stereo and RGB-D cameras to reconstruct the environment to mitigate the issues and use the best of both worlds.

The work of [42] fuses data from RGB-D, thermal, and ZED cameras to obtain reliable point cloud and odometry using the KinectFusion algorithm and its further ICP method plus the ZED VO, filtered through an extended Kalman filter. Results discuss better scenarios than only using one of the methods and more reliable point clouds from the structured light sensor. In [43], the authors used a pair of Kinect cameras to get stereo capabilities from both RGB cameras and the infrared sensor measurements as usual. They used Markov probability functions and GPU parallel processes to predict the best odometry and disparity map from sequential frames. They fused RGB-D and stereo point clouds to complement each other in the scene reconstruction. While both works provide background on the use of sensor fusion, they require computer effort and are not meant to be implemented in an edge-fog environment, but only a local machine, which can distribute the workload and facilitate the interaction with many sensors and the final user.

In order to test this last hypothesis, this work compares the performance of the hybrid sensors approach in a heterogeneous edge environment against the EF, a cutting-edge method in the literature for RGB-D dense SLAM [34].

### 2.2. Heterogeneous Edge Environment

In the last few years, several studies have proposed different classification architectures applied for edge computing platforms. This work considered an edge architecture classification found in the research work presented in [21], where the authors observed that the edge of the network is not clearly defined, and the nodes expected to participate at the edge may vary, and the terminology to describe the edge differs greatly, with the same term being used to define different architectures and functionalities. Therefore, they classified these architectures into three categories based on common features of deployments, and features from different categories can be combined with others. Finally, edge architectures were classified as [21]:

- *Resource-rich servers deployed close to the end-devices:* In this category, the end users connect to resource-rich servers on the network, thus being an option for running edge computing platform. For instance, the authors in [21] cite the cloudlets deployed on WIFI access, which are based on Virtual Machines (VM). They presented an example of the Google Glass, where the data acquired by the sensors are processed on the cloudlet to provide real-time assistance.
- *Heterogeneous nodes at the edge:* This category encompasses different types of resources. As an example, the authors of [21] indicated the use of FOG platforms with heterogeneous nodes processing data for applications.
- *Federation of resources at the edge and centralized data centers:* This category employs edge devices to provide services both locally and in cloud environments. In addition, the services can be distributed throughout the Internet in a flexible infrastructure.

In the last few years, a few efforts have been performed to assist 3D reconstruction and computation approaches, such as in [44,45,46]. In [44], an integrated workflow is proposed where a collaborative online system is used to both reconstruct and publish a Virtual Reality (VR) scene, accessible via Web, from a collection of shared photos. On the other hand, an interesting work presented in [45] states several contributions in the context of robotic map building and dealing with change. The works presented in [46] introduces a complete workflow that allows the generation of 3D models from point clouds of buildings and extracts fine-grained indoor navigation networks from those models, to support advanced path planning for disaster management and navigation of different types of agents.

In this work, we considered a heterogenous edge-fog environment to support a 3D reconstruction case study because it is an interesting architecture to tackle issues where devices, such as cameras and a computer node, are utilized to gather data to compute and process in an accurate fashion to a virtual application.

## 3. The 3D Reconstruction Proposal

**Figure 1:**
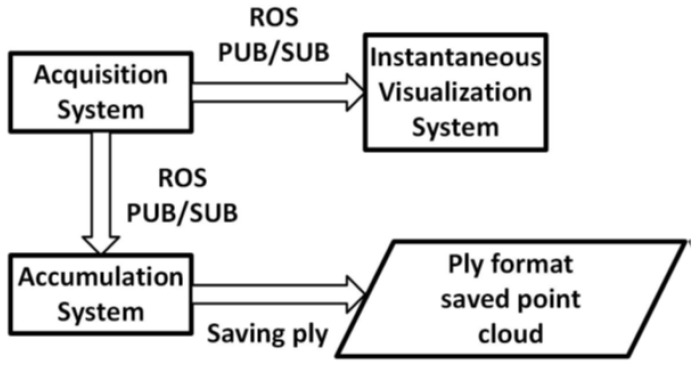
General model of the edge-fog environment.

The 3D reconstruction is a problem solved through SLAM, which means that it is necessary to estimate the sensor motion and register the point cloud in a global model. Every acquired frame generates a point cloud to be fused to this model based on the camera tracked position. To achieve that, we have coupled a stereo camera to an RGB-D camera, connecting them to a computer, configuring the acquisition system. All data acquisition and pre-processing to be delivered to the registration system occur in this node. All communications between processes and devices are done through the publisher-subscriber model utilizing the ROS framework. Figure 1 illustrates a general model of the edge-fog environment. The acquisition system is a worker at the edge, and the accumulation system is the farmer node, which will process the major amount of data.

### 3.1. Acquisition System

**Figure 2:**
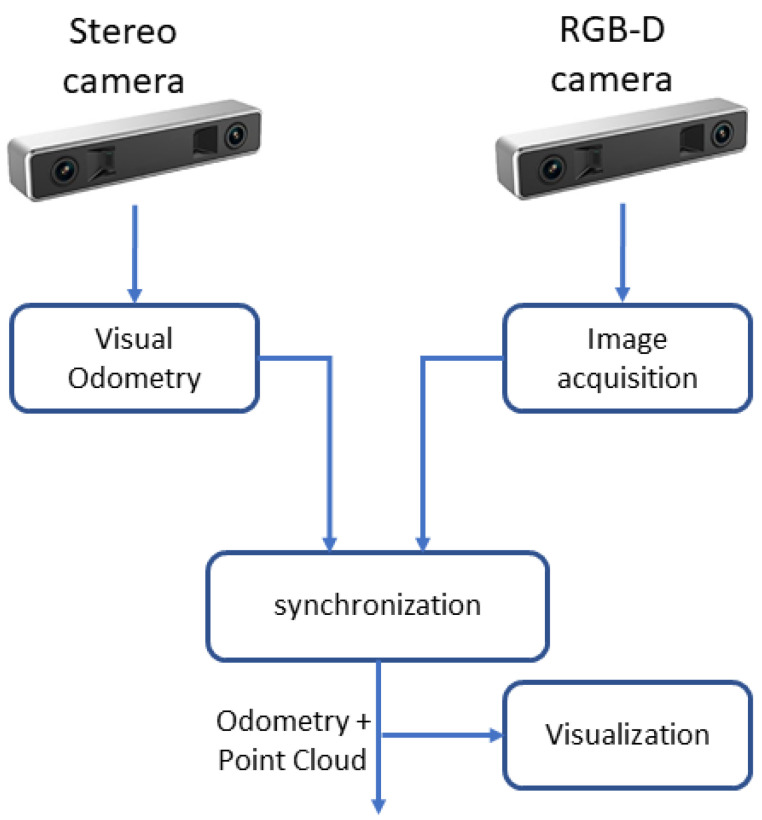
Acquisition system.

The main goal of this edge worker node is to capture images to build the point cloud and estimate the sensor’s movement. Figure 2 shows how the acquisition system works, composed of both cameras as its sensors. The stereo is used to estimate the movement of the sensors through VO, nicely explained in [31], based on the results obtained in [38]. The images are acquired and processed by the ROS package. This publishes the odometry in quaternion format for the rotation and offsets vector for translation, all with respect to the initially acquired frame of the left lens, set as the reference. On the other side, we have another edge device, the RGB-D camera, which is responsible for acquiring and publishing RGB and Depth images through its own ROS node.

Each camera system, i.e., RGB-D and stereo, has its cloud point generation software. In this sense, each device driver’s point cloud will be produced with the original factory accuracy, ensuring that image synchronization is not an issue. This is important to perform because this type of synchronization in software is not simple, and even the usage of a global shutter would be an issue across multiple devices in a variable latency environment.

Visual odometer is also obtained from the ZED camera driver, which ensures 0.35% translation drift and 0.023°/m without loop closure. The differences in odometry will be compensated by the ICP method. It is possible to argue that over time these values will increase. However, the ICP transformation matrices could also be stored in such a case.

The central node, illustrated in Figure 2, is an important element for the project. This is the place where the point cloud generation and synchronization with its respective odometry take place. After that, the data are published in separate topics, making it available for subscription for the viewer and for the accumulation system. Its operation is detailed in Section 3.2.

### 3.2. Accumulation System

First, it is essential to notice that each camera has internal shutter synchronization, meaning that the point clouds are generated independently by the RGB-D and stereo cameras’ systems. In this sense, there is no need to perform any external image synchronization. This is important because performing this type of synchronization without global shutter availability is complex and usually leads to poor results.

**Figure 3:**
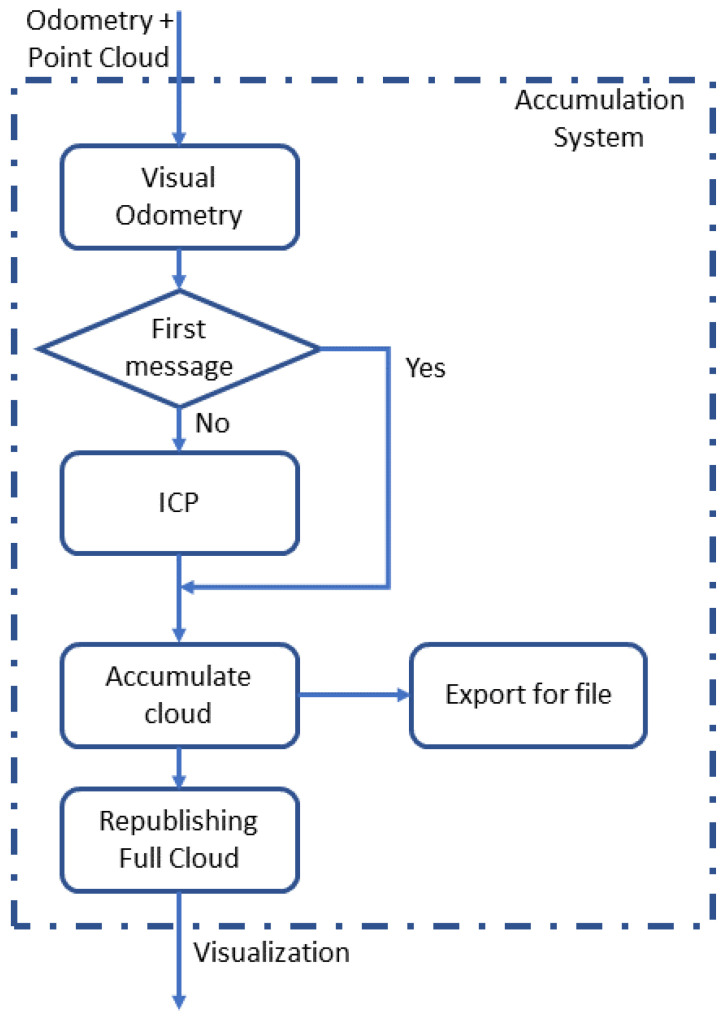
Accumulation approach.

The accumulation system is responsible for processing a large amount of data. Figure 3 provides an idea behind the process. It first subscribes to the ROS topics to get the synchronized odometry and point clouds. Next, it must check whether it is the first message or not to start the accumulation. If it is the first, the received point cloud is accumulated directly and taken as reference. Otherwise, the clouds are passed to an ICP algorithm.

The ICP process was developed using the Point Cloud Library (PCL). The process uses Random sample consensus (RANSAC) applied to the incoming point cloud to find the transformation that produces the proper transformation. The odometry is used as an initial guess to discover the best rigid transformation that fits the new cloud to the last entry [47]. Since odometry is used as an initial guess, the distance among the clouds should be minimal, thus increasing the likelihood of success in the RANSAC method. The fitness score produced by the ICP is outputted for the user. However, there is no additional guarantee that sensors’ outputs are adequately aligned.

The alignment explained, despite being simple, is quite effective. Once the cloud is aligned, the last cloud entry is stored and used at this point instead of the whole accumulated one. This is performed to avoid the increase in processing time when the last one gets larger. Once the rigid transformation is obtained, it is applied to the points of the new cloud to be added to the global model (accumulated cloud).

### 3.3. Heterogeneous Edge-Fog Environment

**Figure 4:**
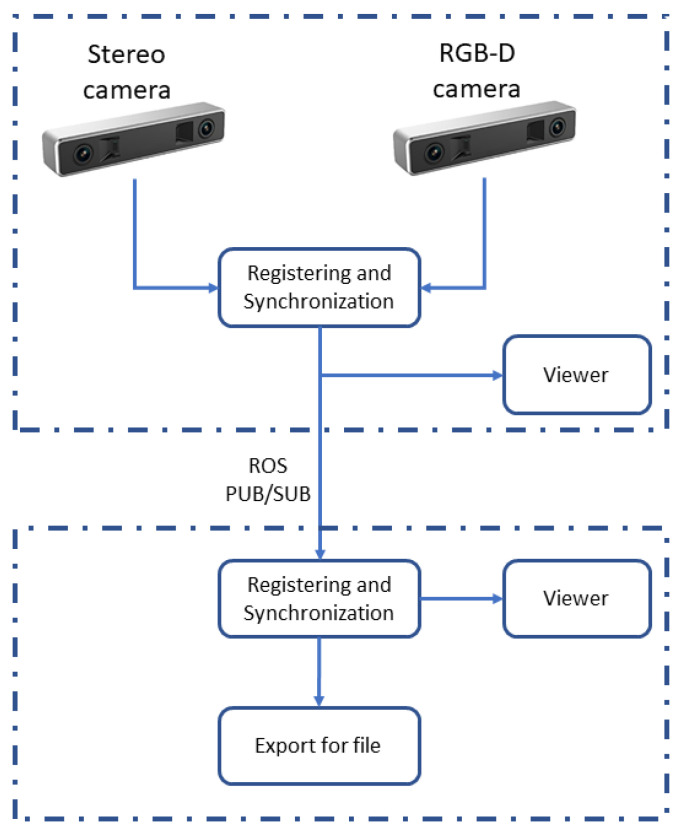
Components of the heterogeneous edge-fog environment.

After establishing the previous components, the edge-fog paradigm is well defined. The acquisition system is the worker at the edge, with a light amount of data to process, providing information to be densely processed by the farmer node. Figure 4 shows two scenarios, called machines I and II. Edge devices, i.e., stereo and RGB-D cameras, process their task, and send the results to a local central node, compounding the Machine I.

Meanwhile, Machine II is characterized as the fog node, which can receive data from several different edge nodes. Adopting this edge-fog architecture, the main goal was to divide the workload, taking the more massive processes to be done far from the acquisition. Therefore, it was possible to determine whether the task is feasible or not. In addition, it is possible to implement the acquisition as an embedded system. This is an important element for future applications, such as 3D reconstructions with a larger amount of data or even inspections with drones.

## 4. Experimental Scenarios and Results

**Figure 5:**
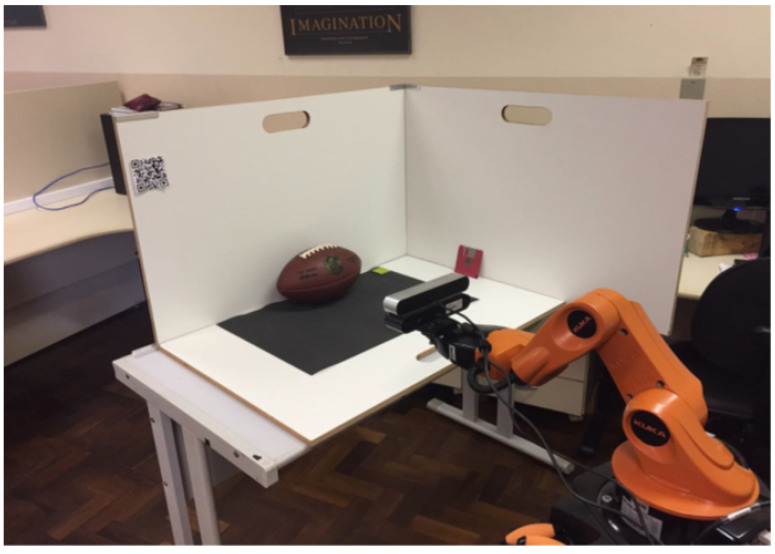
Test scenario.

In this section, we present experimental scenarios, where the edge devices are characterized by different types of cameras in terms of hardware, software, and functions. Therefore, fog environments were formed through different heterogeneous edge devices. It is also important to mention the diverse software from each device, which reinforces a heterogeneous ecosystem. Since the presented work has a considerable number of steps, several different tests were required for evaluation. To achieve that, scenarios were developed, using a robotic arm to generate controlled paths, and having the end-effector pose used as ground truth. Figure 5 shows the primary environment test scenario. The Kuka YouBot robot is supporting two different cameras with different software packages.

It is important to report that the environment material used for the development and evaluation of the scenarios is comprised of:

- StereoLabs ZED stereo camera.
- Orbbec ASTRA RGB-D camera.
- Machine I was a computer Core i7-7700 CPU 3.60 GHz with 16 GB of RAM memory and an NVIDIA GeForce GTX 1050 Ti.
- Machine II was a computer Core i7-6820 CPU 2.70 GHz with 16 GB of RAM memory and an NVIDIA GeForce GTX 1070.
- A Kuka YouBot robot, was utilized as a robotic arm to support the two cameras.
- An American football ball used to be reconstructed in a prepared scenario.

In addition to the physical equipment, we utilized the following ROS packages:

- Package ZED_ROS_wrapper [48].
- Package Astra_camera [49].
- Package Openni2_launch [50].

### 4.1. First Scenario—Path Format Evaluation

**Figure 6:**
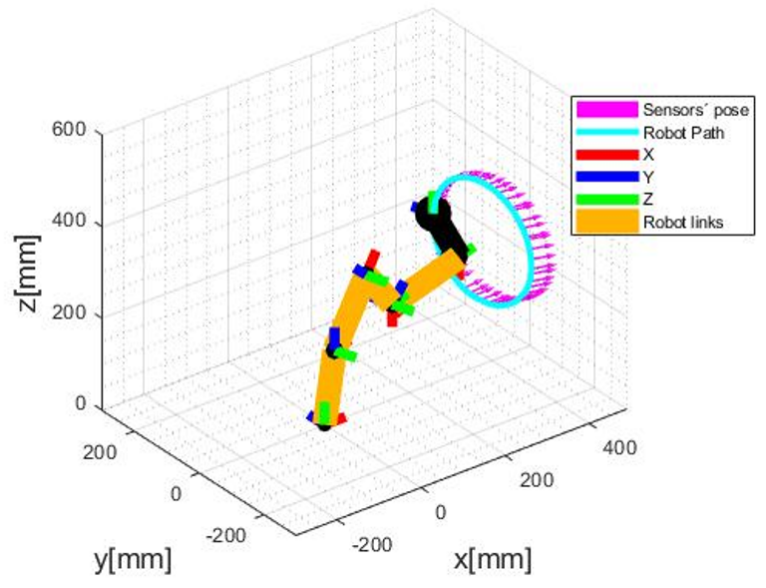
Circular path, in blue, and sensor’s poses in magenta.

**Figure 7:**
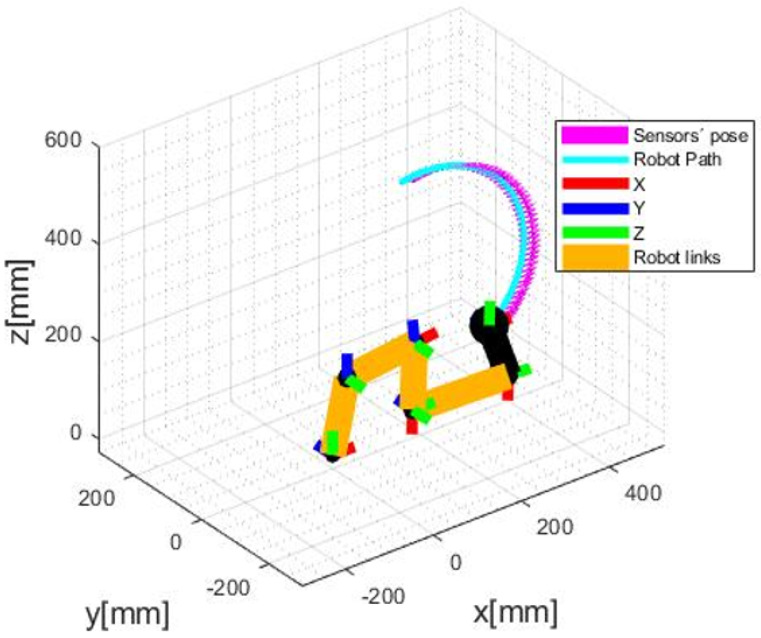
Arc-shape path with varying depth measures.

In this scenario, two different trajectories to be performed by the robot and followed by the odometry system were defined. We are interested in evaluating if the VO is capable of tracking the sensors in curved paths. In the effort to program paths, it was first necessary to obtain the robot’s forward kinematics. To achieve this goal, the Denavit-Hartenberg algorithm was applied. Once with the forward kinematics, it was possible to get the inverse one, used to set a path to be followed, and receive the joint angles to be applied in the robot. With that done, we set a circular and an arc-shaped trajectory, being the first to evaluate with a static depth and the other with varying depth. The odometry measurement was successful in both cases, as shown in Figure 6 and Figure 7. However, it is noticed that there is an error among the arrows and the circular path performed by the robot. This leads to the next scenario, an evaluation of this error.

### 4.2. Odometry Error Evaluation

In this scenario, the main goal is to quantify the error between the odometry and the robot’s path. To achieve that, we synchronized the VO and the robot’s end-effector readings (considered as the ground truth in the scenario), taking into consideration the distance between the gripper and the stereo camera’s left lens. In that way, six measures were taken to be evaluated for the circular trajectory and seven for the arc-shape one. The errors were calculated by taking the differences between each coordinate in every position. The results are presented in Table 1 and Table 2 ahead, presenting the difference between the VO and the ground truth, the average *μ*, and standard deviation *σ* for each coordinate.

The errors found comparing the VO with the ground truth were in a millimeter order of magnitude, agreeing with [38], which was considered a good initial guess for the ICP algorithm prior to point cloud accumulation. However, the error exists, and its effects in the point cloud have to be analyzed.

### 4.3. Point Cloud Construction Analysis

By using the proposed edge-fog architecture, it is possible to have several algorithms running comitantly in different fog nodes focusing on enhancing quality but without losing performance. This approach’s advantage is demonstrated in a 3D reconstruction problem with a featureless white background that will be tested and analyzed by using different typologies.

**Figure 8:**
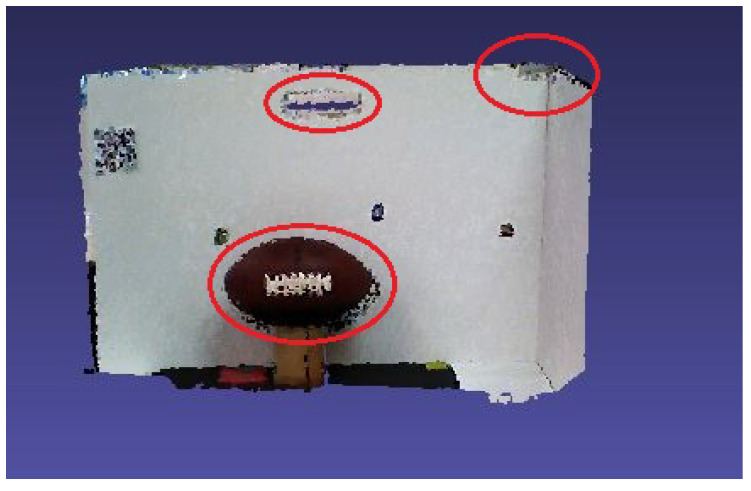
Registered point cloud with only VO adjustment.

**Figure 9:**
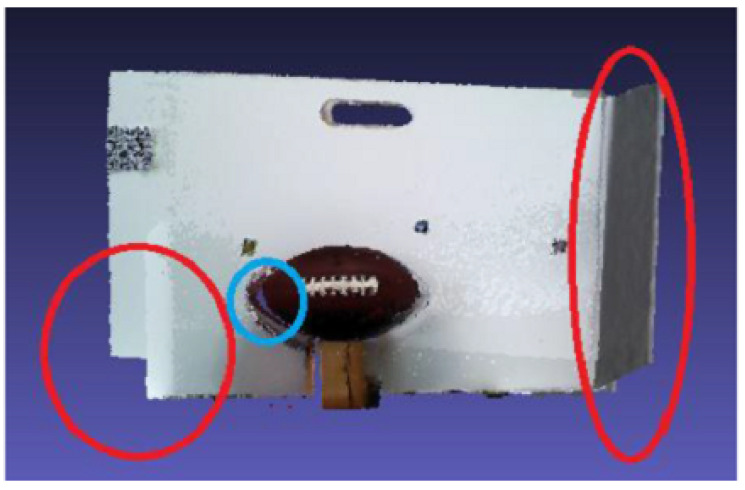
Registered point cloud with elastic fusion adjustment.

**Figure 10:**
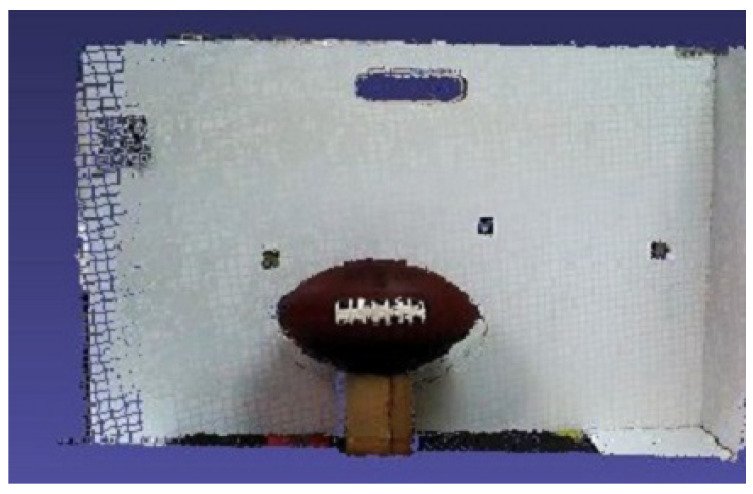
Registered point cloud with ICP adjustment, using VO result as the first guess.

Regarding the comparison, the first configuration is a simple point cloud accumulation using the Visual Odometry Adjustment. The result is shown in Figure 8 and demonstrates that the white panel and the ball were distorted during the process. At the second configuration, the EF method was used, as a fog node, in the same scenario representing a state-of-the-art Dense SLAM solution. The results are presented in Figure 9 and can be noticed that, although the instantaneous point clouds were smooth, with high quality in detail due to its loop closures and deformation, it also had tracking problems, generating a considerable deformation in the registered point cloud, marked in red. The blue circle shows the problem of misalignment between depth and color frames. These results reinforced the hybrid sensor application. The model quality is improved thanks to the RGB-D camera. The biggest problem occurred due to the sensor tracking, which happened although the high processing cost and could be noticed qualitatively. Finally, a serial composition between the VO and ICP algorithms was tested, and the result is shown in Figure 10. It is possible to see better registering adjustment and visual smoothness.

These scenarios have demonstrated that it is possible to use different algorithms, change the information flow, and impact the final result, but not in the final performance.

### 4.4. Edge-Fog Environment Analysis

In this section, we discuss the edge-fog as an infrastructure architecture framework. Therefore, we show evaluations in two dimensions. The first approach was related to the previous reconstruction scenarios and their execution times inside the edge-fog environment compared to an ordinary LAN. The other dimension concerns the challenges with the heterogeneous software packages. A 3D reconstruction experiment illustrates the first test, considering the previous circular path, reconstructing a point cloud of a million points with the distributed system with two machines, and with only one machine system using a local communication. The process was executed ten times for each system. Table 3 shows the obtained results, where one can see that it is possible to use the distributed system at the expense of processing time. It occurs due to the message exchanging time through a LAN network being slower than when locally processed. However, the results have shown a significant reduction in RAM use by 400 MB, which reinforces that the application in an embedded system is possible.

More details about the experiments can be found in the videos posted on the following links for the circular [51] and arc-shaped path [52], respectively: The second dimension was a tacit experience concerning programming the heterogeneous software from the different edge nodes. Challenges were found in the edge camera nodes, where several required dependencies from each software exist in the programming development. It is not straightforward to gather pieces from those heterogeneous edge nodes for them to interoperate. References [48,49,50] are important pillars to support a software development for these IoT devices as a unique environment. Therefore, our empirical experiments indicated, through these heterogeneous edge node environments, issues related to programming IoT software systems compared to conventional systems architectures.

### 4.5. Manual Movement Track Experimentation

**Figure 11:**
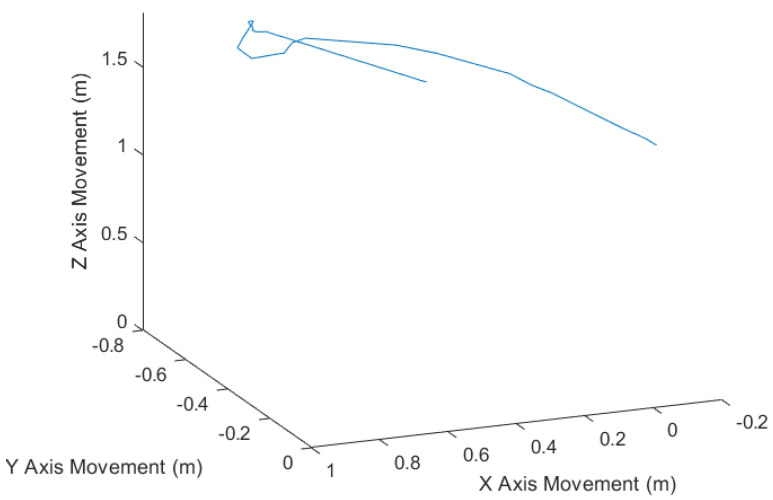
Manual camera movement.

A second test was carried out to demonstrate the architecture operation in a more realistic environment. In this test, the camera was manually moved along a path around the object to be reconstructed. Figure 11 shows the odometry movement.

**Figure 12:**
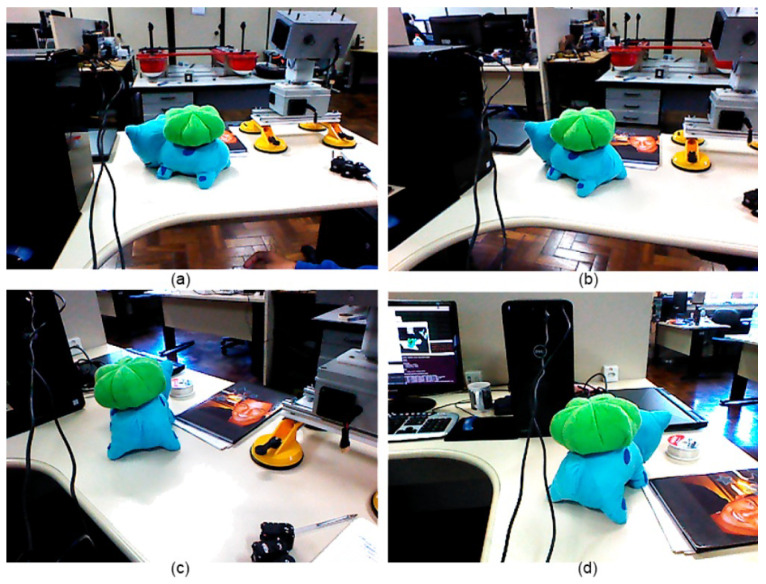
Captured images. (**a**) initial; (**b**) middle; (**c**) middle; (**d**) end.

**Figure 13:**
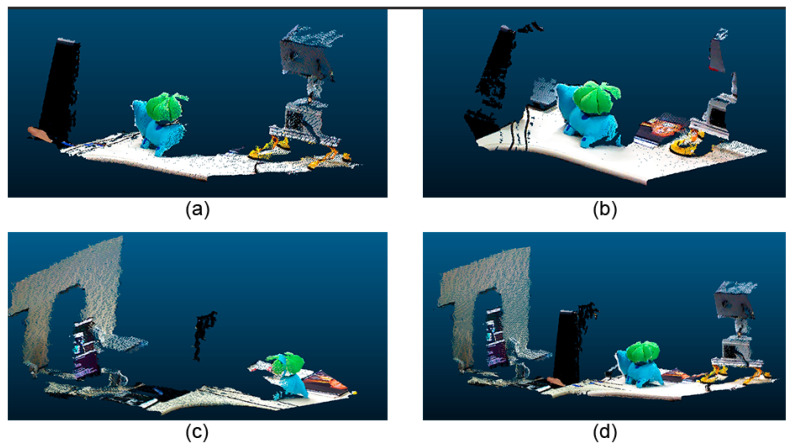
Reconstructed model. (**a**) initial; (**b**) middle; (**c**) middle; (**d**) end.

A complete reconstruction of the inspected object was carried out using the proposed model. Figure 12 presents some RGB images from various angles. The reconstructed model is shown in Figure 13. Note that Figure 13a–c are the point clouds of the RGB camera. Figure 13d is the output of the method, which is the cloud of combined points. As it can be seen, it was effective in combining clouds using the odometry provided by the stereoscopic camera.

In each reconstructed point cloud, there are 214,054 points resulting in approximately 10 MB. As in the previous experiments, two tests were performed: in the first one, the processing was carried out entirely in Edge and the second one shared between Edge and Fog. These results are shown in Table 4 and Table 5, respectively. In this case, the processing time was evaluated considering the processing time of each sample received by the camera, and the edge/fog considering the time for processing each sample in the fog. The CPU usage was sampled on average across all samples. A 100 MB ethernet network was used so that latency did not significantly interfere with processing times.

Note in the data that the application of shared processing was able to reduce the total processing time, still improving the computational load at the edge, allowing more activities to be performed, or even less energy consumption.

### 4.6. Latency and Throughput Analysis

Considering a point cloud with NP points, where three positions represent each point, one for each axis, and variable C_c_ for the number of color channels, (i.e., one channel for luminance, two channels for luminance and matrix, and three for luminance matrix and saturation), we can estimate the size of an uncompressed point cloud size by Equation (1), where *f* represents the frame rate the data type of the position variable (e.g., float or double), and the number of bits of the color channel is 8:

(1) $$Cloud_{Size}=N_{p}\cdot f\dot{(}3\cdot P_{T}+C_{c}\cdot 8)$$

**Figure 14:**
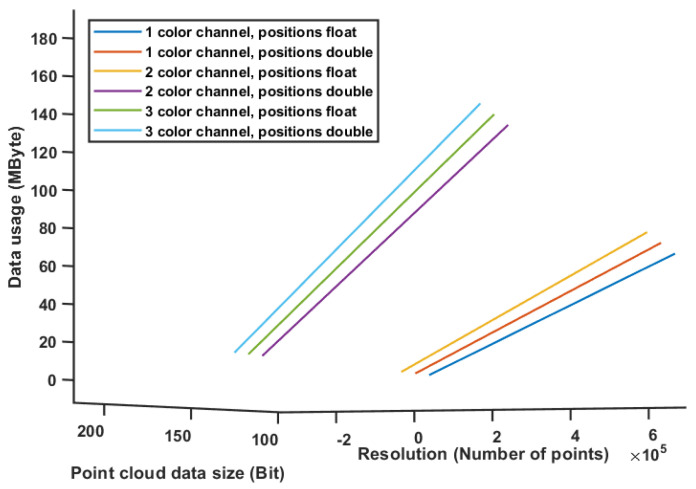
Data usage requirements for uncompressed point cloud in accordance with the number of points and datatype.

Using this formula for a fixed frame rate of 1, and varying sizes of the point cloud, from 256 × 256 up to 1280 × 720, we can plot the data usage required from a network, as shown in Figure 14. As can be noticed, the data usage requirement can grow fast for a fully colored cloud in a reasonable resolution.

It is possible to estimate the impact of latency in the number of sensors being used for a few specific situations. Using a round trip time in ms (RTT) and a given maximum size segment of data (MMS) and considering that there are no lost packets, we can estimate the maximum throughput using Equation (2):

(2) $$Throughput=\frac{MSS}{\left(RTT/1000\right)}$$

**Figure 15:**
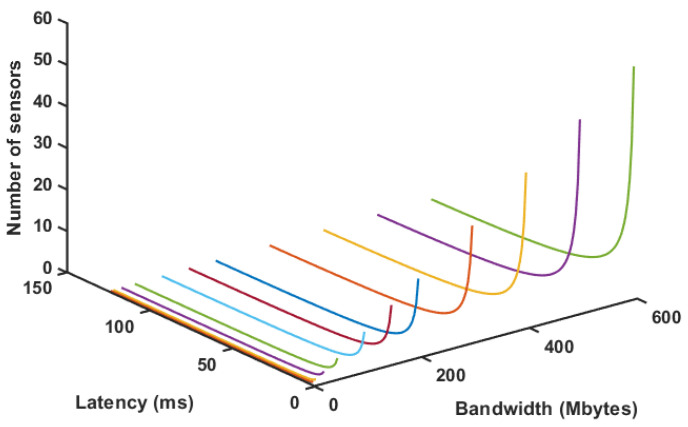
Number of sensors for varying latency and bandwidth for 256 × 256 point clouds.

**Figure 16:**
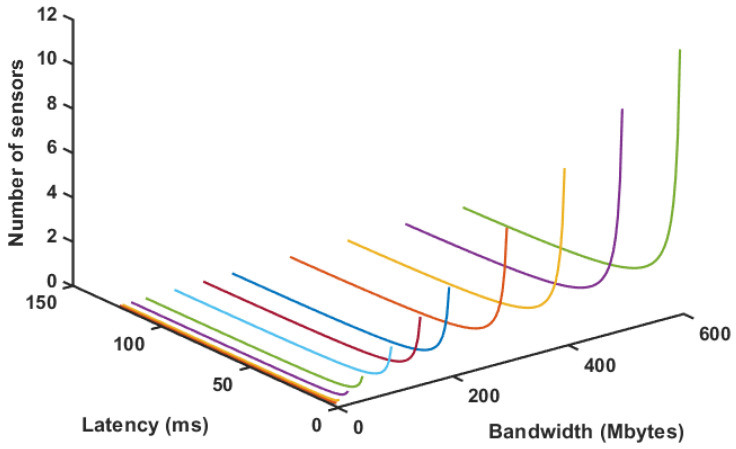
Number of sensors for varying latency and bandwidth for 640 × 480 point clouds.

Then, combining Equations (1) and (2) for a fixed set of conditions, it is possible to estimate the number of possible sensors simultaneously. Figure 15 shows this situation for a fixed MSS of 64 kb and 3 color channel cloud with 256 × 256 points. Figure 16 shows the same situation for a cloud with 640 × 480 points. Despite the different number of sensors, it is possible to notice that these drop dramatically if latency increases significantly. This behavior is expected, once latency tends to limit the amount of data transmission and point clouds are very intense in this regard. This only increases the requirement for a fog node to optimize data if further transmission is required. It is also possible to notice that, with a robust router, or network cables, it is possible to use this configuration between fog and edge with quite a few sensors.

## 5. Conclusions and Future Work

In this paper, we presented a new hybrid approach for 3D reconstruction based on stereo visual odometry allied with the RGB-D dense point cloud. It is also presented as a solution for embedded system applications, separating the acquisition from the registration processes in a heterogeneous edge-fog distributed system. The hybrid sensor approach guaranteed reasonable odometry information, allowing the ICP algorithm to refine it for the point of cloud fusion. Results obtained for the tested trajectories have overcome an Elastic Fusion fused point cloud, which had a duplication due to sensor tracking problems when going through circular paths.

Heterogeneous edge-fog infrastructure results have shown the possibility of applying the acquisition step at the edge, raising future inspection applications with terrestrial robots, or even unmanned aerial vehicles. This feature is reinforced due to ROS framework use, which simplifies the system installation and grants a scalability degree to the system.

As future work, it is in the plans to tackle issues and limitations found during our experiments, for instance adopting new features available to edge-fog environments such as block chain methodologies, and demonstrate by using real applications that need these approaches.

## Figures and Tables

**Table 1:** Position error for the circular path (mm).

Points	P1	P2	P3	P4	P5	P6	*μ*	*σ*
X	0.0002	1.3045	−1.4295	−6.0600	−7.7983	−0.4640	−2.4079	3.6527
Y	−0.1291	1.3241	1.8884	−2.5900	−5.9095	−5.7577	−1.8623	3.4441
Z	0.1530	0.6897	2.5990	2.3022	4.0891	7.2546	2.8479	2.5784

**Table 2:** Position error for the arc-shape path (mm).

Points	P1	P2	P3	P4	P5	P6	P7	*μ*	*σ*
X	−0.2842	4.8284	5.8949	6.7364	7.5845	7.0788	5.4866	5.3322	2.6531
Y	0.0854	0.8135	1.0923	0.2270	−0.8132	−2.0117	−2.7256	−0.4760	1.4413
Z	0.2930	2.4786	6.2703	10.0553	12.5111	12.6021	13.4857	8.2423	5.2964

**Table 3:** Processing time for local (L) and edge-fog (E–F) distributed environment.

Time (s)	T1	T2	T3	T4	T5	T6	T7	T8	T9	T10	Av.	*σ*
**Local**	30.0	34.0	40.0	33.0	34.0	33.0	34.0	32.0	37.0	38.0	34.5	3.0
**Edge**	82.7	77.4	86.1	87.9	83.0	82.8	87.9	80.9	84.6	85.3	83.9	3.2
**Fog**	27.3	32.6	22.9	22.1	28.0	21.2	26.1	21.1	22.4	21.7	24.5	3.8

**Table 4:** Fully edge processing.

Local Processing	Time	%CPU	Network Usage
**Edge**	360 ms	60%	0%

**Table 5:** Shared edge/Fog processing.

Local Processing	Time	%CPU	Network Usage
**Edge**	100 ms	10%	88%
**FOG**	100 ms	10%	88%
